# Clinical Effect of the Traditional Japanese Herbal Medicine “Goreisan” on Water Balance in Patients With Severe Acute Pancreatitis

**DOI:** 10.7759/cureus.63103

**Published:** 2024-06-25

**Authors:** Hiroomi Tatsumi, Masayuki Akatsuka, Hiromitsu Kuroda, Satoshi Kazuma, Yoshiki Masuda

**Affiliations:** 1 Department of Intensive Care Medicine, Sapporo Medical University School of Medicine, Sapporo, JPN

**Keywords:** fluid management, intravascular volume, water balance, goreisan, severe acute pancreatitis

## Abstract

Background: Since severe acute pancreatitis (SAP) involves inflammatory mediators produced by local inflammation of the pancreas that trigger a systemic inflammatory response, intensive fluid management is required to maintain hemodynamics in the early stages of the onset of SAP. Goreisan is considered to have a diuretic effect in a state of excess water and an antidiuretic effect in a state of dehydration, regulating water balance in both directions. We investigated the clinical effects of Goreisan on water balance in SAP patients.

Patients and methods*:* SAP patients admitted to our ICU within 72 hours of being diagnosed with SAP were divided into two groups: the Rikkunshito group (before October 2015) and the Goreisan group (after November 2015). Cumulative volume of fluid infusion, urine, fluid removal by CHF, nasogastric tube drainage, and water balance from day 1 to day 5 of ICU admission.

Results: Thirty patients were included. The median age was 57 (40-69) years, and 21/30 (70%) were male. The prognostic factor score in Japanese criteria for acute pancreatitis was 5.5 (3.3-7). Of the thirty patients, 14 were in the Rikkunshito group, and 16 were in the Goreisan group. There were no differences in the cumulative volume of fluid infusion, urine, fluid removal by CHF, or nasogastric tube drainage from day 1 to day 5 of ICU admission between the two groups. However, the cumulative water balance from day 1 to day 5 of admission was 4,957 ± 6,091 mL in the Rikkunshito group, whereas it was lower in the Goreisan group at 498 ± 3,918 mL (P = 0.023).

Conclusion: Our study showed that Goreisan administration in patients with severe acute pancreatitis might improve water balance in the early phase of onset. Early administration of Goreisan at the onset of severe acute pancreatitis may regulate fluid movement between capillaries and interstitium and alleviate fluid overload due to water refill.

## Introduction

Severe acute pancreatitis (SAP) is an endogenous disease in which inflammatory mediators produced by local inflammation of the pancreas trigger a systemic inflammatory response [1]. Severe acute pancreatitis causes organ disorders such as respiratory failure and acute kidney injury, requiring mechanical ventilation management and continuous kidney replacement therapy (CKRT) [2]. Additionally, in severe acute pancreatitis, decreased gastric peristalsis occurs due to local inflammation of the pancreas. To provide early enteral nutrition, which is important in critically ill patients, we placed a nasojejunal tube and administered Rikkunshito, which is effective in improving gastric peristalsis.

Furthermore, in severe acute pancreatitis, systemic capillary permeability increases due to the overproduction of inflammatory mediators, leading to fluid movement into the interstitium and a decrease in intravascular volume [3]. Therefore, large amounts of fluid exchange, up to several liters per day, are required in the early phase of severe acute pancreatitis to maintain hemodynamics. If sufficient diuresis cannot be obtained due to acute kidney injury when the diuretic phase (period in which leaked water returns to the blood vessels) following the oliguric phase occurs, CKRT may be needed for fluid management. Difficulty managing fluids often leads to various organ disorders such as heart failure, respiratory failure, intestinal edema [4], and abdominal compartment syndrome [5,6].

Goreisan, a traditional Japanese herbal medicine, is one of the hydrating agents that improves local edema (the maldistribution of fluid) caused by inflammation. Goreisan is reported to be effective in preventing the recurrence of chronic subdural hematoma [7] and treating lymphedema after gynecological cancer surgery [8]. In addition, the effectiveness of Goreisan for cardiovascular diseases has been reported, and recently, randomized clinical trials have begun to administer Goreisan for heart failure [9], making it a drug that is attracting attention. We have been using Goreisan since November 2015 in the hope of improving the systemic edema associated with acute-stage severe inflammatory disease, and we also administer it in the early phase of severe acute pancreatitis.

We investigated the clinical effects of Goreisan on volume of fluid infusion, urine, fluid removal by CKRT, and water balance regarding capillary permeability and systemic edema associated with severe acute pancreatitis.

## Materials and methods

Study design

This retrospective study was performed at the ICU of Sapporo Medical University Hospital (Sapporo, Japan). The study protocol conformed to the ethical guidelines enshrined in the Declaration of Helsinki and was approved by the Research Ethics Committee of Sapporo Medical University Hospital (approval number 332-92). Informed consent was waived because this study was a retrospective observational study. An information disclosure document about this study was created and made available to the study patients via the website of the hospital ICU, guaranteeing the opportunity for study patients to refuse.

Patients and setting

The subjects were SAP patients admitted to our ICU within 72 hours of being diagnosed with SAP during the period from January 2007 to July 2021. Patients who were transferred to general wards within four days and those younger than 18 years were excluded. The study patients were divided into two groups: the Rikkunshito group (administered to improve decreased gastric peristalsis due to SAP before October 2015) and the Goreisan group (after November 2015).

Patient characteristics, including age and sex, acute physiology and chronic health evaluation (APACHE) II score and sequential organ failure assessment (SOFA) score at admission, etiology of pancreatitis, prognostic factor score for acute pancreatitis [10], treatments including continuous hemofiltration (CHF) for fluid removal and management of inflammatory mediators, and drugs administered, were evaluated retrospectively. The cumulative volume of fluid infusion, urine, fluid removal by CHF, nasogastric tube drainage, and water balance from day 1 to day 5 of ICU admission, ventilator-free days (VFD), and ICU-free days (IFD) for 28 days were also evaluated.

Statistical analysis

Categorical variables and continuous variables of the patient characteristics are presented as the number (%) and median (interquartile range, IQR), respectively. Urine volume, fluid removal volume by CHF, nasogastric tube drainage volume and water balance from ICU admission to day 5 are presented as the mean ± standard error (SE). The two groups were compared using the χ2 test for categorical variables and the Mann-Whitney U-test and unpaired t-test for continuous variables. All probability values were two-tailed, and P-values less than 0.05 were considered statistically significant.

## Results

**Figure 1:**
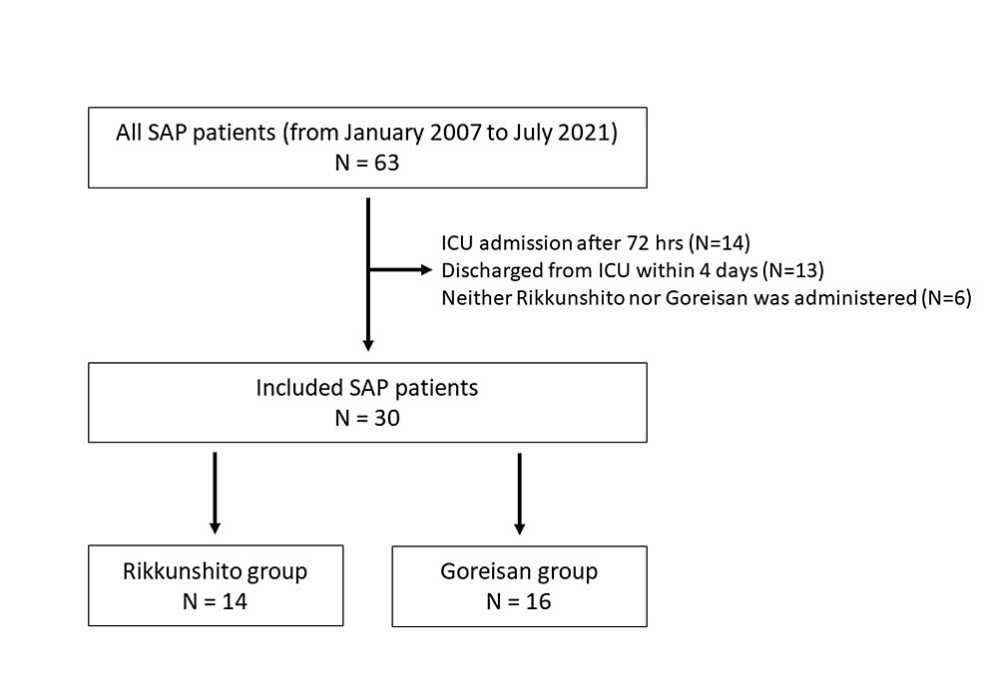
Flow diagram of this study. SAP: severe acute pancreatitis; ICU: intensive care unit.

The characteristics of the patients are listed in Table 1. Thirty patients were included (Figure 1). The median age was 57 (40-69) years, and 21/30 (70%) were male. As indicators of the severity of the illness, the prognostic factor score for acute pancreatitis, the APACHE II score, and the SOFA score were 4 (2.25-5), 15 (11.3-20.8), and 5.5 (3.3-7), respectively. Among the etiologies of pancreatitis, alcoholic was the most common (36.7%), followed by post-endoscopic retrograde cholangiopancreatography (ERCP; 26.7%), gallstones/common bile duct stones (13.3%), and idiopathic (13.3%). Mechanical ventilation was performed on 25 patients (83.3%) and CHF on 28 patients (93.3%). All patients received protein inhibitors and enteral nutrition. VFD and IFD were 21 (12-23) and 17 (7-21), respectively. Only one patient (3.3%) died in the ICU.

**Table 1:** The characteristics of the patients. APACHE: acute physiology and chronic health evaluation; SOFA: sequential organ failure assessment; ERCP: endoscopic retrograde cholangiopancreatography; EUS: endoscopic ultrasonography; CBD: common bile duct; ICU: intensive care unit.

		All patients	Rikkunshito group	Goreisan group	P-value
		n=30	n=14	n=16	
Male, n (%)		21 (70)	11 (78.6)	10 (62.5)	0.576
Age (years)		57 (40–69)	58 (44–72)	57 (34–63	0.450
APACHE II score		15 (11.3–20.8)	15 (13.3–23)	16.5 (9.75–18.8)	0.370
SOFA score		5.5 (3.3–7)	6 (3.3–7.8)	5.5 (3.8–6.3)	0.503
Cause of pancreatitis, n (%)					0.860
	Alcohol	11 (36.7)	3 (21.4)	8 (50.0)	
	Post ERCP/EUS	8 (26.7)	4 (28.6)	4 (25.0)	
	Gallstone/CBD stone	4 (13.3)	2 (14.3)	2 (12.5)	
	Ideopathic	4 (13.3)	3 (21.4)	1 (6.3)	
	Hypertriglyceridemia	2 (6.7)	1 (7.1)	1 (6.3)	
	Ischemia	1 (3.3)	1 (7.1)	0 (0.0)	
Prognostic factor score		4 (2.25–5)	4.5 (3.25–5)	4 (2–5)	0.517
Treatment, n (%)					
	Ventilator	25 (83.3)	11 (78.6)	14 (87.5)	0.870
	Continuous hemofiltration	28 (93.3)	14 (100.0)	14 (87.5)	0.525
	Intraarterial infusion therapy	10 (33.3)	6 (42.9)	4 (25.0)	0.518
	Proteolytic enzyme inhibitor	30 (100.0)	14 (100.0)	16 (100.0)	1.000
	Selective digestive decontamination	6 (20.0)	6 (42.9)	0 (0.0)	0.005
	Early enteral nutrition	30 (100.0)	14 (100.0)	16 (100.0)	1.000
Ventilator free days		21 (12–23)	21 (6.5–24)	21 (17–22)	0.548
ICU free days		17 (7–21)	15 (0–22)	17 (14–20)	0.527
Mortality, n (%)					
	ICU mortality	1 (3.3)	1 (7.1)	0 (0.0)	0.467
	28 days mortality	3 (10.0)	2 (14.3)	1 (6.3)	0.586
	90 days mortality	4 (13,3)	3 (21.4)	1 (6.3)	0.315

Of the thirty patients, 14 were in the Rikkunshito group, and 16 were in the Goreisan group. Selective digestive decontamination (SDD) was administered to 42.9% of the patients in the Rikkunshito group but not in the Goreisan group (P = 0.005). Except for SDD, there were no significant differences between the two groups in patient characteristics and severity, causes of pancreatitis, treatments, VFD, IFD, or prognosis.

**Figure 2:**
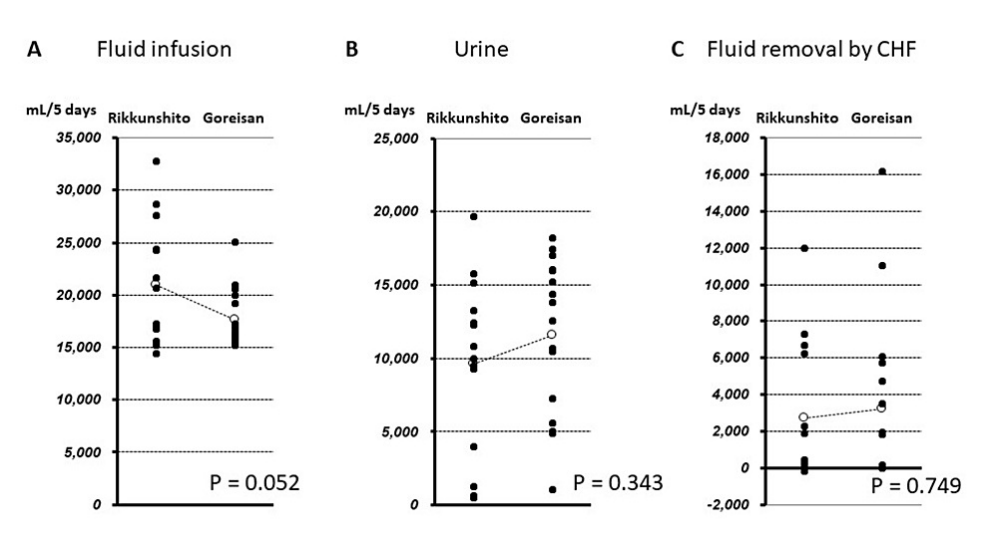
Difference of cumulative volume in five days between two groups. CHF: continuous hemofiltration.

**Figure 3:**
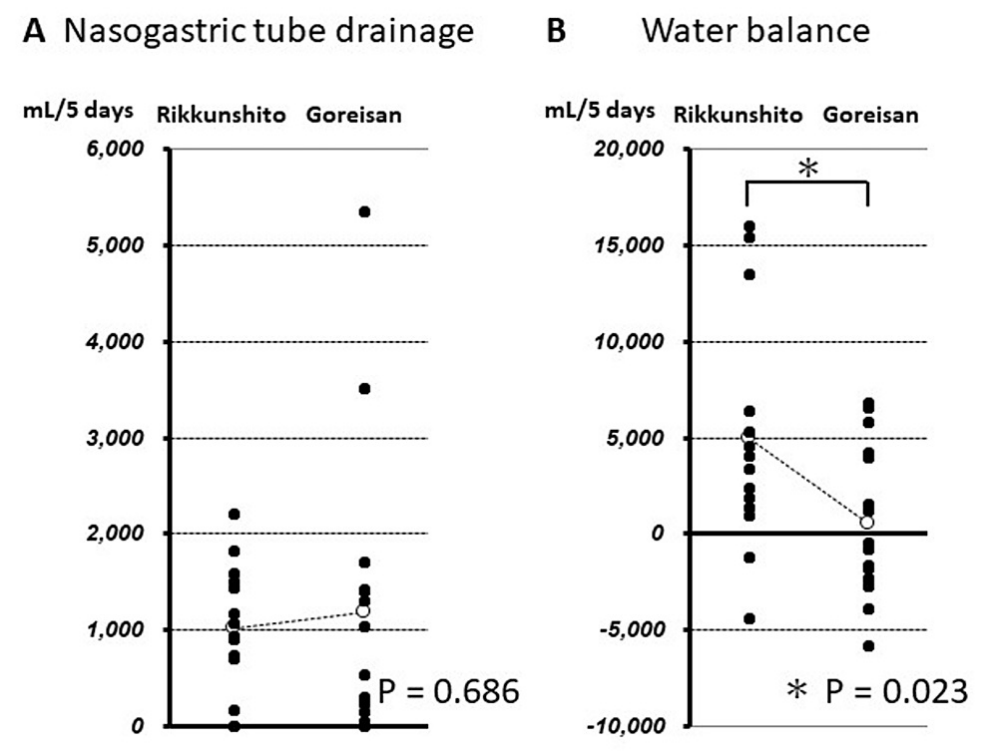
Difference of cumulative volume in five days between two groups. *The cumulative water balance from day 1 to day 5 in the Goreisan group was significantly lower than that in the Rikkunshito group (P = 0.023).

The cumulative volume of fluid infusion from day 1 to day 5 of ICU admission was 20,928 ± 5,777 mL in the Rikkunshito group and 17,656 ± 2,730 mL in the Goreisan group, and although there was no significant difference, it tended to be smaller in the Goreisan group (P = 0.052) (Figure 2A). The cumulative volume of urine was 9,605 ± 5,966 mL in the Rikkunshito group and 11,598 ± 5,348 mL in the Goreisan group, with no difference between the two groups (P = 0.343) (Figure 2B). Similarly, fluid removal by CHF and nasogastric tube drainage were 2,699 ± 3,796 mL and 1,014 ± 667 mL in the Rikkunshito group, and 3,208 ± 4,693 mL and 1,184 ± 1,431 mL in the Goreisan group, respectively (P = 0.749 and P = 0.686, respectively) (Figure 2C, 3A). However, the cumulative water balance from day 1 to day 5 of admission in the Goreisan group was lower than that in the Rikkunshito group (498 ± 3,918 mL vs. 4,957 ± 6,091 mL, respectively, P = 0.023) (Figure 3B).

## Discussion

This study is the first to examine the administration of Goreisan in the early phase of the onset of severe acute pancreatitis. Although there was no difference in cumulative volume of urine or fluid removal by CHF alone from day 1 to day 5 after ICU admission, regardless of whether Goreisan was administered, the cumulative water balance was significantly reduced by Goreisan administration. It has been suggested that Goreisan may alleviate fluid movement between capillary vessels and the interstitium in patients with severe acute pancreatitis.

In response to increased capillary permeability early in the onset of severe acute pancreatitis, hemodynamics will collapse unless fluid resuscitation is performed. However, excessive fluid infusion causes severe systemic edema, and cardiac stress causes serious conditions such as heart failure, pulmonary edema, pleural effusion, and ascites [11]. Therefore, in the early stages of the onset of severe acute pancreatitis, intensive systemic management is required to restore the fluid balance to an even level by restricting the fluid as much as possible to maintain hemodynamics and recovering thereafter the administered fluid by diuresis and fluid removal by CKRT [12]. If the patient is weaned from the ventilator before the fluid balance has returned to an even level, hemodynamic and respiratory conditions will worsen, leading to a prolonged period of mechanical ventilation and an ICU stay. However, in severe acute pancreatitis, a large amount of body fluid moves from the capillaries to the interstitium, and it takes time to refill the fluid, resulting in a long-term decrease in intravascular volume and insufficient volume of urine and fluid removal by CKRT.

In Kampo medicine, local edema (uneven distribution of water) due to inflammation is considered fluid stagnation. Goreisan is composed of the herbal medicines Alisma tuber (Sengoku), Atractylodes lancea rhizome (Cangshu), Polyporus sclerotium (Bolivia), Poria sclerotium (Boului), and Cinnamon bark (Cinnabar) [8], and is a typical Japanese herbal prescription “hydric agent” for treating water stagnation. Unlike diuresis in Western medicine, fluid regulation with Goreisan is considered to have a diuretic effect in a state of excess water and an antidiuretic effect in a state of dehydration, regulating water balance in both directions. One of the mechanisms of action of Goreisan has been suggested to be the inhibition of water permeability through inhibition of water channels by inhibiting aquaporin 4 upregulation [13,14], and Atractylodes lancea rhizome and Polyporus sclerotium are considered to be involved. Poria sclerotium and Alisma tuber are also said to have fluid-regulating effects [5]. There are various aquaporin families throughout the body, and Goreisan has been reported to have effects on several of them. Goreisan is thought to have the effect of regulating vascular permeability and moving fluid to its proper location via aquaporins. Therefore, Goreisan may be effective against increased not only for severe acute pancreatitis but also for increased capillary permeability and systemic edema in other inflammatory diseases such as sepsis.

The limitations of our study are listed below. First, the study design was a retrospective study at a single institution, which introduces inherent biases and limits the generalizability of our findings. Second, the sample size was small, which may impact the statistical power and generalizability of the findings. Third, until October 2015, we administered Rikkunshito to patients with severe acute pancreatitis to improve the decreased gastric peristalsis associated with pancreatitis. In November 2015, Rikkunshito was switched to Goreisan, with an emphasis on suppressing the movement of water into the interstitium due to increased capillary permeability. Therefore, there may be some historical changes in other treatments as well. Other biases may be present, so caution is advised in generalizing the results of this study. In the future, large-scale multicenter randomized controlled trials will be able to exclude the effects of these factors and provide insight into the usefulness of Goreisan administration in severe acute pancreatitis.

## Conclusions

Our study showed the potential benefit of Goreisan administration in patients with severe acute pancreatitis, particularly in the early phase of onset. The improvement in water balance suggests that Goreisan may play a role in regulating fluid movement between capillaries and the interstitium. Goreisan is thought to be effective not only for severe acute pancreatitis but also for serious conditions such as sepsis, in which vascular permeability changes markedly.
